# Influence of physician years on urological journal publication productivity among Japanese urologists

**DOI:** 10.1186/s40064-016-3696-x

**Published:** 2016-11-29

**Authors:** Naoya Niwa, Eiji Kikuchi, Kazuhiro Matsumoto, Akira Miyajima, Mototsugu Oya

**Affiliations:** Department of Urology, Keio University School of Medicine, Shinanomachi 35, Shinjuku-ku, Tokyo, 160-8582 Japan

**Keywords:** Authorship, Gender, Physician years, Post-graduate year, Urology, Publication

## Abstract

**Purpose:**

To evaluate the urological journal publication productivity of Japanese urologists based on their physician years.

**Methods:**

All original articles written by Japanese urologists and published from Japanese institutions in 6 primary urological journals between 2010 and 2014 were reviewed (N = 529 articles).

**Results:**

The median post-graduate years of the first and corresponding authors of all articles were 16 and 21 years, respectively. The publication productivities of the first and corresponding authors peaked from 11 to 15 and both 16–20 and 21–25 post-graduate years, respectively. In 187 publications in which the first and corresponding authors were different, first and corresponding author publication productivities peaked from 11 to 15 and 21–25 post-graduate years, respectively. In 342 publications in which the first author served as the corresponding author, first author publication productivity peaked from 16 to 20 post-graduate years. Of all articles examined, 130 (24.6%) were written by young urologists who had obtained their national medical license not less than 11 years ago. Only 0.9% (5/529) of all articles were written by female Japanese urologists, who account for 5.0% (332/6,649) of all Japanese Board Certified Urologists.

**Conclusions:**

The present study revealed that from 11 to 15 post-graduate years was the most productive time for Japanese urologists as the first author of urological publications and also that the role in manuscript preparation changed with increases in physician years. These results provide an insight into reconstructing future post-graduate training and educational urological programs in Japan.

## Background

Medical research and academic publications in urology are essential not only for the development of urology as a scientific discipline, but also the improvement of patient treatment and care. Clinical urologists have less time and financial incentive to dedicate to academic activity concurrent with clinical practice, and fewer skills and less knowledge to conduct research and write scientific articles. However, a large number of scientific articles written by urologists have been published in several journals including urological journals, and the number of papers will increase in the future.

Limited information is available on the authorship of articles written by urologists. One study evaluated the gender of authors and revealed that female urologists produced manuscripts at a rate that exceeded their number in the urology field (Weiss et al. 2012). Publication productivity and the authorship of urology residents have also been evaluated (Hellenthal et al. 2009; Finkelstein et al. 2015), and the findings obtained suggest that significant research time is needed during residency for publication productivity.

The length of time in clinical practice is one of a physician’s characteristics because it affects the quality of medical care provided. A systematic review suggested that physicians in practice for longer may be at risk of providing lower quality care (Choudhry et al. 2005). On the other hand, surgical experience gained with time has a positive impact not only on operation times and the incidence of surgical complications, but also oncological outcomes (Atug et al. 2006; Thompson et al. 2014). However, few studies have investigated the association between the length of time in clinical practice and scientific publication productivity in the urology field.

In this study, we examined the characteristics of urologists who published scientific articles in urology journals, and evaluated the urological journal publication productivity of Japanese urologists based on their physician years.

## Methods

We selected 6 primary urological journals: *The Journal of Urology*, *BJU International*, *Urologic Oncology: Seminars and Original Investigations*, *World Journal of Urology*, *International Journal of Urology*, and *Urology*, to represent urological literature. All articles published in each journal between 2010 and 2014 were reviewed. Only articles from institutions in Japan and original articles were included in our present study. We excluded editorials, reviews, letters to the editor, case reports and other articles without an abstract.

In each article, the journal name, published year, title of the article, gender of the first and corresponding authors, post-graduate years of the first and corresponding authors, and the institutions to which the corresponding authors belong were recorded. The genders and the post-graduate years of the authors were determined as follows. An Internet search was performed using the Google™ search engine in order to find Japanese names. By using the public physician database provided by the Ministry of Health, Labor and Welfare (https://licenseif.mhlw.go.jp/search/), the genders and physician years of the first and/or corresponding authors registered with the Japanese Registry of Physicians were determined. In Japan, most physicians register with the Japanese Registry of Physicians immediately after obtaining their national medical license; therefore, the year when the author registered was regarded in the present study as the same year that the author obtained their national medical license.

We defined young urologists as those that had obtained their national medical license not less than 11 years ago. Non-young urologists were defined as those that had obtained their national medical license 11 or more years ago. In Japan, after the completion of two years of mandatory and globally post-graduate clinical training defined by law, physicians join the Japanese Urological Association (JUA) and start comprehensive training in clinical urology in order to become urologists. After the completion of four years of training in clinical urology and success in JUA examinations, physicians obtain a Japanese Board Certified Urologist license. After the completion of an additional five-year educational course for urological instructors defined by the JUA, physicians obtain a Japanese Urology Board Certificated Instructor license. Therefore, physicians require at least 11 years of post-graduate clinical training and education to obtain a Japanese Urology Board Certificated Instructor license.

The variables of the different groups examined were compared using the Chi squared test, Mann–Whitney *U* test, and an analysis of variance, as appropriate. All statistical analyses were performed with the SPSS version 22.0 statistical software package (IBM, Armonk, NY).

## Results

### Publication characteristics

A total of 605 original articles were published in the 6 journals from Japanese institutions between 2010 and 2014. Among these, one article was retracted and 75 were written by authors other than Japanese urologists; therefore, the remaining 529 articles were included in the analysis. The profiles of these articles are shown in Table 1. More than one third of all articles were published in the *International Journal of Urology*, which is an official journal of the JUA. The second most frequent urological journal was *Urology* (21.7%).

**Table 1:** The profile of 529 articles written by Japanese urologists

	Total (N = 529)
Journal, n (%)	
J Urol	80 (15.1)
BJU Int	89 (16.8)
Urol Oncol	33 (6.2)
World J Urol	20 (3.8)
Int J Urol	192 (36.3)
Urology	115 (21.7)
Publication years, n (%)	
2010	118 (22.3)
2011	92 (17.4)
2012	93 (17.6)
2013	108 (20.4)
2014	118 (22.3)

*J Urol* The Journal of Urology, *BJU Int* BJU International, *Urol Oncol* Urologic Oncology: Seminars and Original Investigations, *World J Urol* World Journal of Urology, *Int J Urol* International Journal of Urology

### First and corresponding author distribution in 529 articles over 5 years

**Fig. 1:**
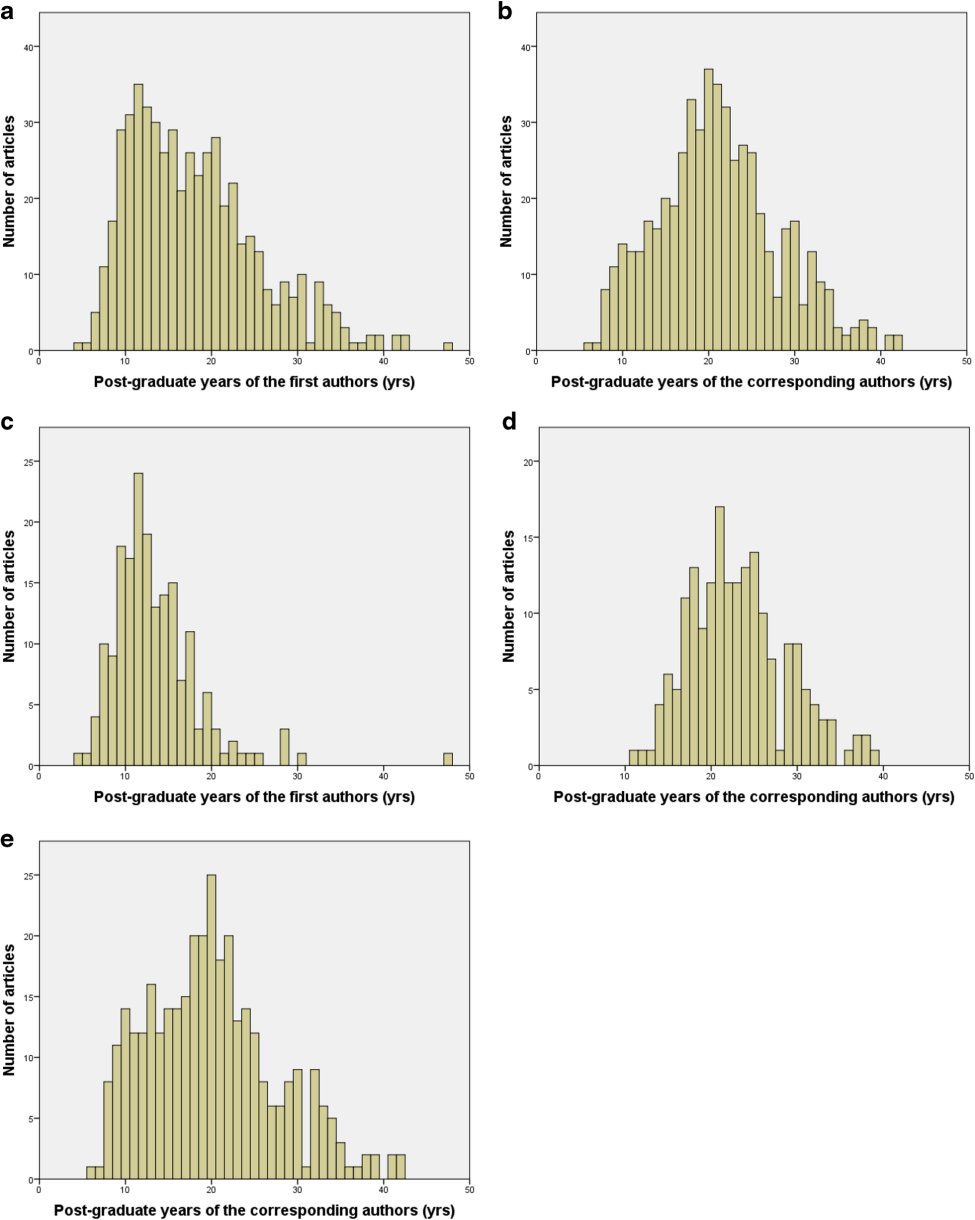
Distribution of publications from Japanese urologists in 6 urology journals with post-graduate years during the year of article publication between 2010 and 2014. **a** First authors of all 529 articles, **b** corresponding authors of all 529 articles, **c** first authors of 187 articles in which the first and corresponding authors were different, **d** corresponding authors of 187 articles in which the first and corresponding authors were different, and **e** corresponding authors of 342 articles in which the first author served as the corresponding author

The distribution of the 529 publications from Japanese urologists in the first and corresponding authors’ post-graduate years during 5 years of article publication is shown in Fig. 1a, b, respectively. The median post-graduate years of the first and corresponding authors of the 529 articles were 16 and 21 years, respectively (*p* < 0.001). Of the 529 articles, the first and corresponding authors were different in 187 (35.3%) articles, while the first author served as the corresponding author in the remaining 342 (64.7%) articles. The distribution of the 187 publications in the first and corresponding authors’ post-graduate years during the 5 years of article publication is shown in Fig. 1c, d, respectively. Figure 1e shows the distribution of the 342 publications in the corresponding author’s post-graduate years during the 5 years of article publication. The median post-graduate year of the corresponding authors of the 187 articles in which the first and corresponding authors were different was 23 years, which was significantly higher than that in the 342 articles in which the first author served as the corresponding author (20.0 years, *p* < 0.001). Furthermore, the percentage of post-graduate years in the corresponding author of 20 or less years in the 187 articles in which the first and corresponding authors were different was 33.7% (63/187), which was smaller than that in the 342 articles in which the first author served as the corresponding author (195/342, 57.0%, *p* < 0.001).

**Fig. 2:**
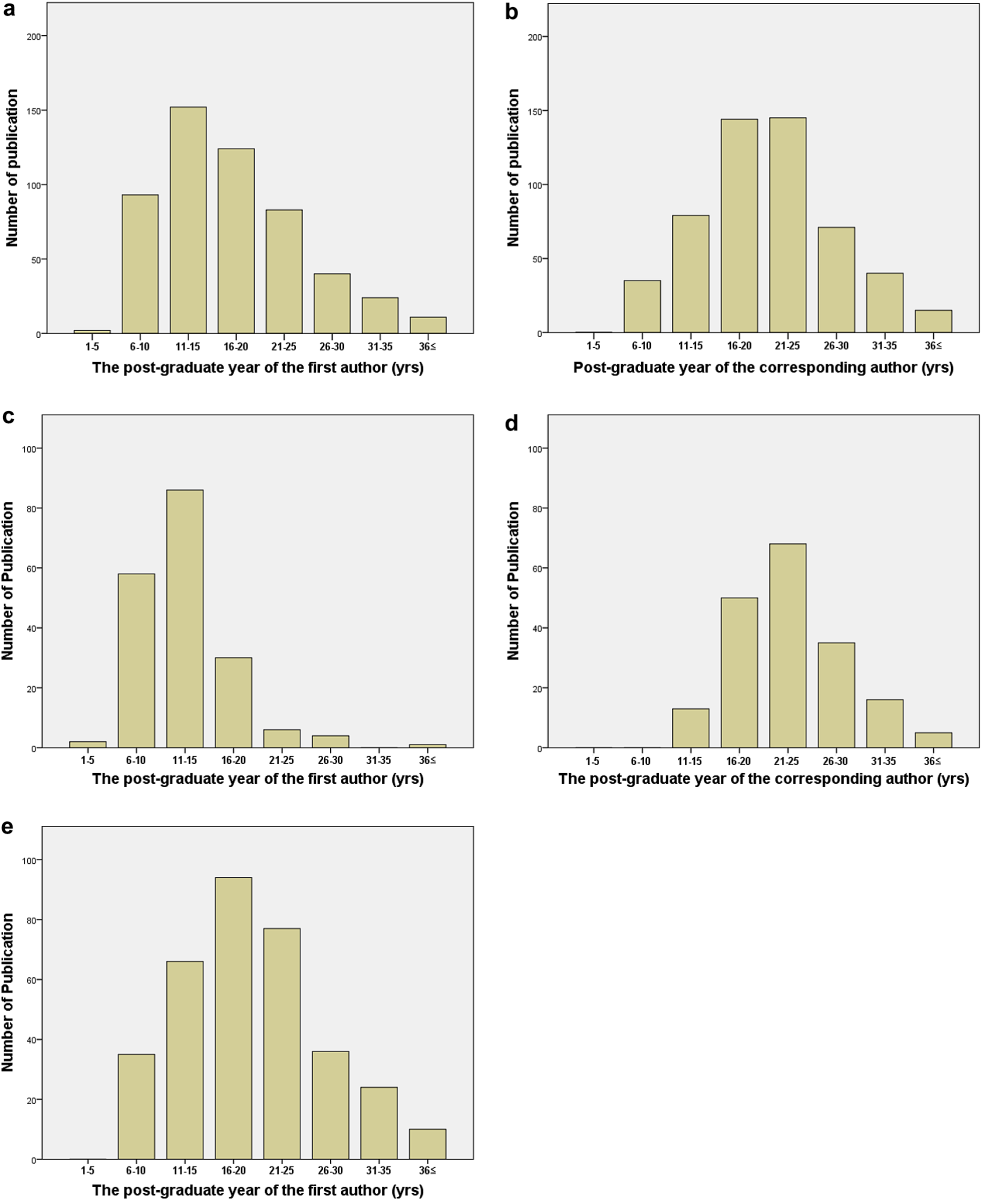
Distribution of publications from Japanese urologists in 6 urology journals in 5-years intervals of post-graduate years during the 5 years of article publication between 2010 and 2014. **a** First authors of all 529 articles, **b** corresponding authors of all 529 articles, **c** first authors of 187 articles in which the first and corresponding authors were different, **d** corresponding authors of 187 articles in which the first and corresponding authors were different, and **e** corresponding authors of 342 articles in which the first author served as the corresponding author

The distribution of publications from Japanese urologists in eight 5-year intervals of author post-graduate years during the 5 years of article publication is shown in Fig. 2. First author publication productivity peaked from 11 to 15 post-graduate years and then gradually decreased (Fig. 2a). Corresponding author publication productivity peaked from 16 to 20 and 21–25 post-graduate years (Fig. 2b). In the 187 publications in which the first and corresponding authors were different, first and corresponding author publication productivities peaked from 11 to 15 and 21–25 post-graduate years, respectively (Fig. 2c, d). In the 342 publications in which the first author served as the corresponding author, first author publication productivity peaked from 16 to 20 post-graduate years (Fig. 2e).

### First author publication characteristics in 529 articles during the 5 years stratified by young and non-young urologists

Among all 529 articles, 130 (24.6%) were written by young urologists and only 7 (1.3%) by young urologists who had obtained their national medical license not less than 6 years ago. We compared author backgrounds between articles written by young urologists and non-young urologists (Table 2). No significant differences were observed in the journal type (p = 0.164) or institution type to which the corresponding author belongs (p = 0.149) between the two groups. The proportion of publications by female urologists was 3.1% in young urologists (4/130 urologists), which was significantly higher than that in non-young urologists (0.3%, 1/399 urologists, p = 0.014). The proportion of publications in which the first and corresponding author was the same was 35.4% in young urologists (46/130 urologists), which was significantly lower than that in non-young urologists (74.4%, 297/399 urologists, *p* < 0.001).

**Table 2:** The comparison of author background of articles between written by young urologists and non-young urologists

	Total (N = 529)	Young (N = 130)	Non-young (N = 399)	*P* value
Journal, n (%)				
J Urol	80 (15.1)	18 (13.8)	62 (15.5)	0.164
BJU Int	89 (16.8)	27 (20.8)	62 (15.5)	
Urol Oncol	33 (6.2)	3 (2.3)	30 (7.5)	
World J Urol	20 (3.8)	7 (5.4)	13 (3.3)	
Int J Urol	192 (36.3)	44 (33.8)	148 (37.1)	
Urology	115 (21.7)	31 (23.8)	84 (21.1)	
Gender, n (%)				
Male	524 (99.1)	126 (96.9)	398 (99.7)	0.014
Female	5 (0.9)	4 (3.1)	1 (0.3)	
Post-graduate years (years)				
Median	16	10	19	NA
Interquartile range	12–22	8–11	15–24	
Institution, n (%)				
University hospital	478 (90.4)	121 (93.1)	357 (89.5)	0.149
Others	51 (9.6)	9 (6.9)	42 (10.5)	
First AU is corresponding AU, n (%)				
Yes	343 (64.8)	46 (35.4)	297 (74.4)	<0.001
No	186 (35.2)	84 (64.6)	102 (25.6)	

*J Urol* The Journal of Urology, *BJU Int* BJU International, *Urol Oncol* Urologic Oncology: Seminars and Original Investigations, *World J Urol* World Journal of Urology, *Int J Urol* International Journal of Urology, *AU* author, *NA* not available

## Discussion

In the present study, we found that the median post-graduate year of the first author in urological publications from Japanese urologists was 16 years and first author publication productivity peaked from 11 to 15 post-graduate years. We also showed that the median post-graduate year of corresponding authors in urological publications from Japanese urologists was 21 years and corresponding author publication productivity peaked from 16 to 20 and 21–25 post-graduate years. Benway et al. (2009) have also examined publishing productivity in urological articles and demonstrated that the *h*-index, which is defined as the number of publications h that have each been cited at least *h* times in published reports, was highly associated with academic standing at the top urology programs in the United States. However, the author’s position in the author’s list of the manuscript could not be reflected by the *h*-index. Our study clearly evaluated the publishing productivity between the first authors and the corresponding author separately in 6 urological articles. To the best of our knowledge, this is the first study to identify differences in publishing productivity between the first and corresponding authors.

Previous studies revealed that publication productivity increased with occupational age, reached a peak at some point during the career, and then declined gradually (Falagas et al. 2008), which was consistent with the results of the present study. In most studies, actual age was regarded as occupational age. Since there is no age limitation to become a physician, actual age does not always represent a physician’s age, namely, the length of time in clinical practice is sometimes not associated with actual age. In our present study, the period between the year when an author registers with the Japanese Registry of Physicians and the year when an article is published is set as the length of time in clinical practice. This measurement is more accurate for estimating the real length of time in clinical practice than actual age.

The post-graduate education and training system for physicians including urologists varies between countries. One study performed in the United States on the academic productivity of residents demonstrated that a 5-year urological residency program produced fewer publications than a 6-year program because of the shorter time dedicated to research, (Finkelstein et al. 2015) Our study revealed that few young urologists produced urological publications during or soon after their residency program in Japan. Only 0.2% (1/529), 0.2% (1/529), 0.9% (5/529), 2.1% (11/529), and 3.2% (17/529) of all articles were written by urologists with 4, 5, 6, 7, and 8 post-graduate years, respectively. The urological residency program in Japan requires 4 years without time dedicated to research, which may have a negative impact on future publication productivity.

The present analysis revealed two interesting results. The publication productivity of female Japanese urologists is extremely low. A bibliometric study to examine authorship gender in *The Journal of Urology* and *Urology* revealed that 16.7% (101/604) of all articles from institutions in the United States were written by female urologists, who account for 6.2% of all urologists (652/10,493) (Weiss et al. 2012). In contrast, our study to examine authorship in 6 general urology journals reveals that only 0.9% (5/529) of all articles from institutions in Japan were written by female urologists, who account for 5.0% (332/6,649) of all Japanese Board Certified Urologists. The exact reason why the publication productivity of female Japanese urologists is extremely low currently remains unknown. Differences in educational programs between Japanese and American urologists have been suggested as one reason. The proportion of female medical students, medical doctors, and urologists is increasing in Japan. Our study also reveals that the proportion of publications by females is significantly higher in young urologists than in non-young urologists. In the future, an educational strategy that provides knowledge on study design and instructions for producing publications is needed in order to enhance Japanese urological practices, particularly for young urologists.

The second interesting result obtained in the present study is that the role in manuscript preparation changes with increases in physician years. As described above, first author publication productivity peaked from 11 to 15 post-graduate years. During this period, the first author conducts research and prepares manuscripts under the supervision of corresponding or other senior authors, and learns the skills of scientific writing. In the next five years, Japanese urologists hold the role of the first and corresponding author. The author’s role includes not only conducting research and preparing manuscripts, but also planning research and responding to any correspondence. During this period, through trial and error, physicians learn how to create and manage scientific publications without strict or educational instructions. In the subsequent five years and thereafter, Japanese urologists shift their emphasis to the role of corresponding author.

There are some limitations to the present study. It is affected by selection bias because articles published in only 6 primary urological journals are examined. These articles does not reflect all urological literature. We did not include *European Urology*, which is the top urological journal, in our analysis because few articles from Japanese institutes have been published in that journal. Furthermore, articles written by Japanese urologists, but published by institutes located in countries other than Japan were excluded from the analysis. Some Japanese urologists study abroad for a few years and engage in research and the preparation of manuscripts in the middle of their careers.

## Conclusions

The present study revealed that from 11 to 15 post-graduate years is the most productive time for Japanese urologists as the first author in urological publications. We also clearly demonstrated that the role in manuscript preparation changes with increases in physician years. These results provide an insight into the reconstruction of future post-graduate training and educational urological programs in Japan.

## Abbreviation

JUA: Japanese Urological Association
